# Nanosheets with High-Performance Electrochemical Oxygen Reduction Reaction Revived from Green Walnut Peel

**DOI:** 10.3390/molecules27010328

**Published:** 2022-01-05

**Authors:** Yifei Zhou, Lei Yan, Junhua Hou

**Affiliations:** 1 School of Physics and Information Engineering, Shanxi Normal University, No. 339 Taiyu Road, Xiaodian District, Taiyuan 030031, China; 219110014@sxnu.edu.cn (Y.Z.); 220110010@sxnu.edu.cn (L.Y.); 2 Extreme Optical Collaborative Innovation Center, Shanxi University, No. 92, Wucheng Road, Xiaodian District, Taiyuan 030006, China; 3 Modern College of Humanities and Sciences, Shanxi Normal University, No. 501 Binhe West Road, Yaodu District, Linfen 041000, China

**Keywords:** biomass carbon, oxygen reduction reaction, N-doped, catalyst, methanol tolerance, stability

## Abstract

The synthesis of metal-free carbon-based electrocatalysts for oxygen reduction reactions (ORR) to replace conventional Pt-based catalysts has become a hot spot in current research. This work proposes an activation-assisted carbonization strategy, to manufacture N-doped ultra-thin carbon nanosheets (GWS180M800) with high catalytic activity, namely, melamine is used as an accelerator/nitrogen source, and walnut green peels biological waste as a carbon source. The melamine acts as a nitrogen donor in the hydrothermal process, effectively enhancing the nitrogen doping rate. The content of pyridine nitrogen groups accounts for up to 48.5% of the total nitrogen content. Electrochemical tests show that the GWS180M800 has excellent ORR electrocatalytic activity and stability, and makes a quasi-four-electron ORR pathway clear in the alkaline electrolyte. The initial potential and half slope potential are as high as 1.01 and 0.82 V vs. RHE, respectively. The GWS180M800 catalyst has a better ability to avoid methanol cross poisoning than Pt/C has. Compared with 20 wt% Pt/C, GWS180M800 has improved methanol tolerance and stability. It is a metal-free biochar ORR catalyst with great development potential and application prospects. This result provides a new space for the preparation of valuable porous nano-carbon materials based on carbonaceous solid waste and provides new ideas for catalyzing a wide range of electrochemical reactions in the future.

## 1. Introduction

With the gradual intensification of global energy and environmental issues, the development and utilization of clean energy have become an inevitable development trend. Fuel cells are considered to be a promising and effective alternative due to their clean, stable, and sustainable characteristics [1,2,3,4]. However, the excessively high overpotential and slow kinetics of the cathode inhibited its large-scale development. In addition, Pt and catalysts, which are considered to have the best catalytic activity, greatly limit their commercial development due to their limited reserves, high prices, susceptibility to methanol cross-effects, and CO poisoning, and poor tolerance [5,6,7]. As a result, it is very important to accelerate the ORR reaction rate and improve the energy conversion efficiency of the battery for developing new energy sources and protecting the environment [8].

Studies have shown that the oxygen reduction electrocatalyst prepared from biomass-derived materials, such as activated carbon [9], enzymes [10], microorganisms [11], and transition metal porphyrin [12], solid organic waste extract [13], has the characteristics of green and easy availability, superior performance, high stability, and high activity, and it has the potential to replace precious-metal-based ORR catalysts [14,15,16,17]. Consequently, biomass-derived materials have drawn great attention from researchers [18,19,20,21,22,23].

According to public data, China’s walnut output was about 3.627 million tons in 2019. In the process of walnut kernel processing, a large amount of green walnut peel is wasted because it is not effectively used. Thus, converting it into high value-added ORR catalyst products has huge environmental and economic benefits. This research will provide an easy-to-implement method to prepare a stable ORR catalyst from renewable waste green walnut hull biomass and provide an innovative strategy for the production of biomass.

In this work, a catalyst synthesis strategy with green walnut skin as the precursor, a simple preparation process, and low cost was proposed. The synthesis process includes a hydrothermal process under melamine doping and then annealing with N_2_. The surface morphology and structural characteristics of the catalysts are manifested by SEM, TEM, Raman, XPS, etc., and further explored the mechanism of catalyst activity improvement. The results show that after activation, a large number of organic pores are formed, a higher specific surface area, and the N doping rate (10.46%) and defects have been significantly improved, which is of great significance to the improvement of catalyst activity. It is worth noting that the catalyst GWS180M800 exhibits high catalytic activity, methanol tolerance, and high stability in alkaline media. The catalyst also shows good activity against acidic ORR. This strategy converting agricultural and forestry wastes into high-value-added products is simple, low-cost, and easy to promote. 

The oxygen reduction reaction in an aqueous solution can generally be carried on through the four-electron pathway and the two-electron pathway. The four-electron pathway directly reduces oxygen to water, while the two-electron pathway has hydrogen peroxide as the intermediate of the reaction. The four-electron pathway is obviously preferable to the two-electron pathway with the reaction intermediate hydrogen peroxide, and the path used for the specific catalyst depends on the type of catalyst [24].

Many articles on non-metallic catalysts have clear explanations of the neutral and alkaline reaction mechanisms of oxygen reduction reactions [25,26]. The reaction mechanism can be summarized as:

Four electronic paths:

(1) $$O_{2}{+2H}_{2}{O+4e}^{-}\to 4{\mathrm{OH}}^{-}\;$$

Two electronic paths:

(2) $$O_{2}{+2H}_{2}{O+2e}^{-}\to {\mathrm{HO}}_{2}^{-}\;$$

(3) $$O_{2}^{-}{+H}_{2}{O+2e}^{-}\to 3{\mathrm{OH}}^{-}\;$$

In an acidic medium, the mechanism can be described as follows:

Four electronic paths:

(4) $$O_{2}{+4H}^{+}{+4e}^{-}\to 2H_{2}O\;$$

Two electronic paths:

(5) $$O_{2}{+2H}^{+}{+2e}^{-}\to H_{2}O_{2}\;$$

(6) $$H_{2}O_{2}{+2H}^{+}\to 2H_{2}\;$$

## 2. Results and Discussion

All electrochemical measurements are completed in electrochemical workstation (Chenhua CHI760E, Shanghai China). The test is carried out under a three-electrode system, with 0.1 M KOH aqueous solution as the electrolyte, with a catalyst-modified glass rotating disc electrode (GC-RDE) as the working electrode, and with platinum wire with 3 M/L KCl solution and the Ag/AgCl electrodes as the counter electrode and the reference electrode, respectively. Firstly, the glassy carbon electrode (GC, diameter 3 mm) is polished with 0.05 mm Alumina powder. Secondly, use an appropriate amount of absorbent cotton dipped in a small amount of absolute ethanol to wipe the surface of the electrode clean. Then, 5 mg of catalyst, 50 μL Nafion solution, 250 μL isopropanol, and 700 μL deionized water are mixed in a 1.5 mL centrifuge tube and sonicated for 1 h with a sonicator to form a catalyst suspension. Finally, take 10 μL droplets of the catalyst suspension on the surface of the electrode and wait for it to dry naturally (the average catalyst loading is 0.25 mg · cm^−2^).

To keep the gas saturated in the solution, pass oxygen into the electrolytic cell for 30 min before testing. Then the ORR performance of the material was tested by cyclic voltammetry (CV) and linear sweep voltammetry (LSV). 

According to the Nernst equation, the potential measured in this article is converted into a reversible hydrogen electrode (RHE) scale

(7) $$E_{VS.RHE}=E_{VS.Ag/AgCl}+E_{VS.Ag/AgCl}^{\theta }+0.059pH\;(25\;{}^{\circ}C)$$

The value of the electron transfer number (n) can be calculated from the slope of the linear fitting line according to the Kentucky-Levich equation:

(8) $$\frac{1}{i}=\frac{1}{i_{l}}+\frac{1}{i_{k}}=\frac{1}{B\omega^{1/2}}+\frac{1}{i_{k}}$$

(9) $$B=0.62nFC_{0}D_{0}^{2/3}v^{-1/6}$$

i is the actual measured current density; $i_{k}$ and $i_{l}$ is the kinetic and limiting diffusion current density; ω is the angular velocity of the disk; n is the total number of electrons transferred in the ORR; F is the Faraday constant (96,485$\;{C\;\mathrm{mol}}^{-1}$); $C_{0}\;$is the oxygen concentration in 0.1 M KOH ($1.2\times 10^{-6}\;\mathrm{mol}\cdot {\mathrm{cm}}^{-3}$); $D_{0}$ is the diffusion coefficient of oxygen in 0.1M KOH ($1.9\times 10^{-5}{\;\mathrm{cm}}^{-2}\cdot s^{-1}$); v is the dynamic viscosity of the electrolyte ($1.0\times 10^{-2}{\;\mathrm{cm}}^{2}\cdot s^{-1}$).

In a 0.1 M KOH solution saturated with O_2_, a rotating disk electrode (RRDE) was used to test the number of electron transfer and hydrogen peroxide yield of the catalyst at 1600 rpm and a scan rate of 50 mV/s. Then use the formulas below to calculate n and $H_{2}O_{2}$ yield [27].

(10) $$n=4\times \frac{I_{d}}{I_{d}+I_{r}/N}$$

(11) $$\%HO_{2}^{-}=200\times \frac{I_{r}/N}{I_{d}+I_{r}/N}$$

When $I_{d}$ is the disk current, $I_{r}$ is the ring current, and  N (N = 0.37) is the current collection efficiency of the Pt ring.

**Figure 1:**
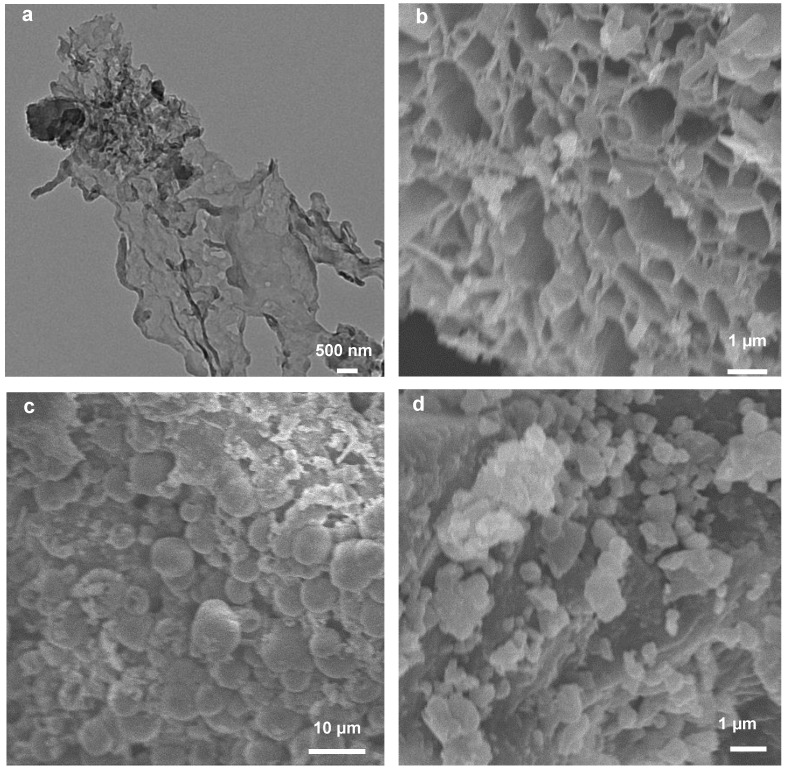
TEM images of GWS180M800 (**a**). SEM images of GWS180M800 (**b**), GWS180 (**c**), and GWS180M800 (**d**).

The scanning electron microscope (SEM) and transmission electron microscope (TEM) images of GWS180M800 are shown in Figure 1a,b. Figure 1c,d show the SEM results of the catalysts GWS180M and GWS800, respectively. It can be seen from the SEM image that the melamine and the walnut green peel will be hydrothermally combined, and the combination will collapse to form a honeycomb porous structure during the high-temperature carbonization process. The SEM images of GWS800 and WS180M800 show a block structure, so melamine has an important influence on the formation of the porous structure of the catalyst. In addition, the TEM image further shows that the prepared WS180M800 becomes thinner, which is consistent with the result of the SEM honeycomb structure.

**Figure 2:**
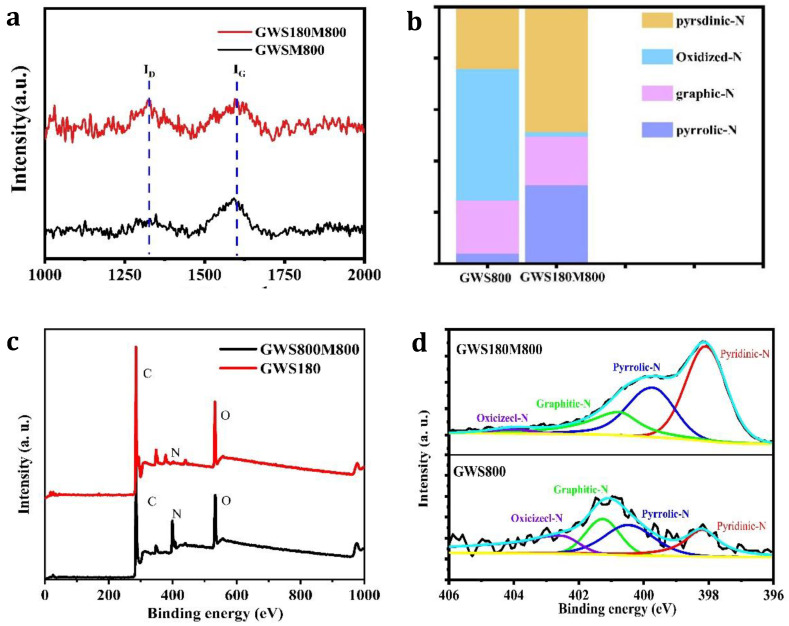
Raman spectra (**a**) of catalysts XPS spectrum (**b**), high-resolution XPS spectrum of N 1s (**c**), 3D bar graphs of the relative content of nitrogen species on the surface of GWS180M800 (**d**).

Raman spectroscopy is used to determine the degree of graphitization of various catalysts prepared under different conditions. The Raman spectrum is shown in Figure 2a. All samples have two significant characteristic peaks: the D peak at about 1320 cm^−1^ corresponds to amorphous carbon and the G peak at about 1590 cm^−1^ corresponds to graphitized carbon. The ratio of the intensity of the D peak to the intensity of the G peak is an important index for evaluating the degree of graphitization of a material [28]. The larger the *I_D_*/*I_G_* ratio, the lower the degree of graphitization, and the greater the degree of defects in the catalyst. It can be seen that the *I_D_*/*I_G_* ratio of the catalyst GWS180M800 increases significantly, which is due to the formation of more defects with the introduction of melamine [29].

To analyze element composition and bonding configuration, we tested and analyzed the X-ray photoelectron spectroscopy (XPS) of all the GWS-X samples. In Figure 2b, the spectrum of GWS-X material shows three peaks. The peak centers are 285, 400, and 532 eV respectively, corresponding to C1s, N1s, and O1s elements [30], which shows that the obtained GWS-X sample still maintains a certain amount of nitrogen element and has an oxygen functional group. Most notably, the N content of GWS180M800 relative to GWS180 has increased significantly from 0.87% to 10.46%, which has a huge effect on the improvement of its catalytic performance. To study the bonding environment of the elements, we analyze the high-resolution XPS spectra of C1s and N1s of the GWS-X series.

According to elemental analysis, the C content of GWS800, GWS180M700, GWS180M800, GWS180M900, GWS180M1000 are 81%, 73.91%, 70.98%, 76.43%, 76.43%, and 79.18%, and the N content is 0.87%, 2.11%, 10.46%, 5%, and 1.28%, which strongly proves that melamine acts as a nitrogen donor in the hydrothermal process, effectively enhancing the nitrogen doping rate (Table S1). Figure 2b shows four characteristic peaks, located in the range of 284.15–284.58, 285.06–286.34, 286.24–287.04, and 289.19–290.18 eV, corresponding to sp^2^ hybridized graphitic carbon C-C/C = C, C-N/C = N bond, C = O, and O-C = O bond. 

Figure 2c is a high-resolution N1s spectrum, showing four characteristic peaks, which are located in the range of 398.10–398.84, 399.54–399.89, 400.33–400.82, and 401.39–402.15 eV [31], which are attributed to pyridine-N, pyrrole-N, graphite-N, and nitric oxide bonds [32]. Furthermore, the atomic percentage of the GWS180M-X sample is quantitatively analyzed by fitting the peak area. The N content of GWS800 is 0.87%, the N content of GWS180M700 is 2.11%, the N content of GWS180M800 is 10.46%, the N content of GWS180M900 is 5%, and the N content of GWS180M1000 is 1.28% (Figure 2d) [32], indicating higher pyrolysis temperature may cause the loss of nitrogen functional groups. Nevertheless, to our surprise, GWS180M800 reveals the highest nitrogen content in the honeycomb carbon structure [33,34,35]. After an in-depth analysis of nitrogen species (Figure 2d), in the GWS180M 700, GWS180M 800, GWS180M 900, and GWS180M1000 samples, pyridine-N and graphite-N accounted for 51.31%, 81.9%, 64.5%, and 13.3% of the total nitrogen components respectively. The GWS180M 800 material contains the highest nitrogen active substance in the GWS180M-X series of samples, which indicates that the catalyst may exhibit excellent electrochemical performance. Thus, the results of XPS can further explain that various N species have been successfully embedded in the carbon skeleton, which will produce a large number of active sites and structural defects [32,36,37].

**Figure 3:**
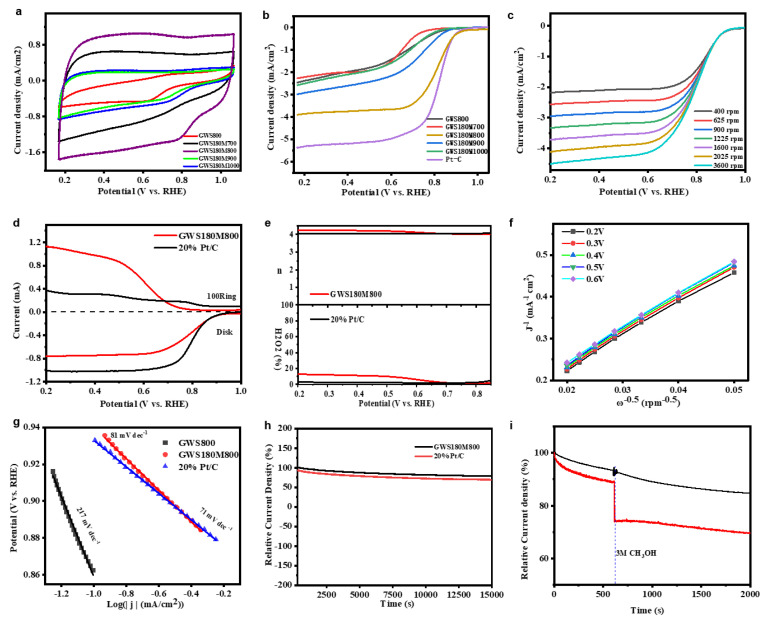
CV curves (**a**) of all catalysts in O_2_ saturated 0.1 M KOH solution at room temperature and scanning rate of 50 mV/s. The LSV curves of GWS-X at an electrode rotation rate of 1600 rpm and a scan rate of 10 mV/s (**b**). The LSV curve (**c**) of GWS180M800 at different speeds from 400 to 2025 rpm corresponds to the K-L diagram (**f**) GWS180M800 from 0.2 to 0.6 V respectively. The RRDE linear scan voltammogram of GWS180M800 and 20 wt% Pt/C in 0.1 M KOH saturated with O_2_, the electrode rotation rate and scan rate are 1600 rpm and 5 mV/s, respectively (**d**). Electron transfer number n (up **e**) and H_2_O_2_ yield (down **e**) calculated from the results of GWS180M800 and 20 wt% Pt/C RRDE measurement. Tafel slope curve spectrum (**g**). The stability curve of GWS180M800 and 20 wt% Pt/C in O_2_-saturated 0.1 M KOH solution, RDE rotation rate of 1600 rpm 15,000 s stability curve (**h**) and methanol measured by chronoamperometric amperometric method Tolerance performance curve (**i**).

To evaluate the electrocatalytic activity of ORR, five carbon-based ORR catalysts are coated on the surface of the GC-RDE electrode respectively and further tested by cyclic voltammetry (CV) and linear sweep voltammetry in a 0.1 M KOH solution saturated with O_2_ Method (LSV). The electrochemical results regarding ORR activity are shown in Figure 3a. It can be found that in the O_2_ saturated electrolyte, all the CV curves of GWS800, GWS180M700, GWS180M800, GWS180M900, GWS180M1000 show ORR peaks clearly, which can be obtained by comparing the peak potential scores of the relative RHE. The ORR activities of the five-carbon catalysts follow GWS180M800 > GWS180M900 > GWS180M700 > GWS180M1000 > GWS800 in the order. What’s more, the LSV curve (Figure 2b) records the saturated KOH solution obtained by O_2_ at a speed of 1600 rpm to further understand the catalytic activity of GWS800, GWS180M700, GWS180M800, GWS180M900, and GWS180M1000. Compared with the GWS800 catalytic electrode with E_1/2_ of 0.70 V with RHE, the electrode catalyzed by GWS180M800 shows better ORR activity when the half-wave potential (E_1/2_) is 0.82 V. In addition, on the GWS180M800 catalyst, the initial potential is 1.01 V, and a higher limiting current density can be obtained, which is comparable to the commercial Pt/C catalyst electrode (20 wt%). These results are in good agreement with the results of CV measurement, and further display the excellent ORR activity of GWS180M800. Due to a large number of defects and the improvement of nitrogen doping efficiency in the pyrolysis process, the catalytic activity of ORR is improved. The high annealing temperature caused a large loss of N content from 10.46% (800 °C) to 1.28% (1000 °C) [38], as demonstrated by XPS. That’s why the value of the limiting current density indicates GWS180M800 > GWS180M900 > GWS180M700 > GWS180M1000 > GWS800. Accordingly, finding the optimal carbonization temperature is very important for preparing carbon material samples with high ORR activity.

Then, Tafel analysis is used to obtain kinetic information. As shown in Figure 3g, the Tafel slope (81 mV dec^−1^) of GWS180M800 is close to the Tafel slope (71 mV dec^−1^) of the reference 20 wt% Pt/C), which indicates that there is a high exchange current density at the interface between the catalyst and the electrode. It is beneficial for practical applications. GWS800 showed a large Tafel slope of 217 mV dec^−1^, indicating a poor ORR dynamic process. These results indicate that the optimal pyrolysis temperature is essential for enhancing electrochemical activity [39]. Generally, a lower annealing temperature (700 °C) will lead to insufficient carbonization, which is harmful to the formation of high graphitic carbon, and a higher calcination temperature (1000 °C) will cause the accumulation of graphite layers to increase. Higher pyrolysis temperature will always lead to the loss of N content. As proven by XPS, GWS180M800 exhibits the most excellent ORR performance in terms of initial potential and limiting current density.

To further evaluate the ORR reaction mechanism, the catalyst was coated on a rotating ring disk electrode (RRDE), and the LSV curve was measured in a 0.1 M KOH solution saturated with O_2_. The rotation speed was from 200 to 2000 rpm. With the rotation speed increasing, the limiting current density also increases correspondingly, and the high rotation speed will cause the diffusion distance to be shortened. At the same time, the LSV curve shows that the process may be a four-electron reaction. The measurement result is shown in Figure 3d. Both GWS180M800 and 20 wt% Pt/C showed lower ring current density, indicating that less H_2_O_2_ was detected on the ring electrode, which means that the catalyst has higher catalytic activity. In Figure 3e, based on the ring current and disk current data of RRDE, we calculated the electron transfer number (n) and H_2_O_2_ yield through formulas. GWS180M800′s n is 3.88~3.95, and the yield of H_2_O_2_ in the potential range of 0.2~1.0 V is slightly higher than that of 20 wt% Pt/C. proves that GWS180M800 is a quasi-four-electron reaction and its catalytic activity is close to Pt/C. This corresponds to the calculation result of the K-L (Figure 3f) equation [40,41].

The stability of GWS180M800 and Pt/C catalysts was also tested by current-time chronograph measurement (Figure 3h) [42,43]. With time passing by, their current density has decreased. However, the descending speed of GWS180M800 is slower than that of Pt/C, as shown in Figure 3h. After the 15,000 s test, S5a, GWS180M800, and 20 wt% Pt/C still maintained 77.3% and 72.5% of the initial current respectively. It shows that GWS180M800 has more long-term stability than the 20 wt% Pt/C catalyst has.

For ORR catalysts, the actual fuel cell must consider its resistance for cross effects. As shown in Figure 3i, in the O_2_ saturated KOH solution injected with 3.0 M methanol, a sharp oxidation current of 20 wt% Pt/C in the i-t curve is observed. Under the same conditions, the cathode of GWS180M800 changes slightly. Add 5 mL of anhydrous methanol to the 0.1 M KOH electrolyte saturated with O_2_ at 600 s, and the currents of GWS180M800 and 20 wt% Pt/C at 2000 s are 80.4% and 70.1% of the initial current respectively. This clearly shows that the prepared GWS180M800 catalyst has a better ability to avoid methanol cross poisoning than Pt/C has. Compared with 20 wt% Pt/C, GWS180M800 has improved methanol tolerance and stability. It is a metal-free biochar ORR catalyst with great development potential and application prospects.

On the basis of the above-mentioned data, the GWS180M800 are indeed highly active toward the ORR with quite positive half-wave potentials and large limiting current densities in alkaline media and outperform most of the other equivalent benchmarks and Pt-based electrocatalysts (Table 1). The material has quite high ORR catalytic performance, which is mainly due to two aspects. First, the abundant mesoporous structure can greatly improve the mass/electron transfer efficiency and provide a large number of exposed active catalytic sites. Secondly, the best N element doping can produce charging defects, adjust the surface polarity of the carbon skeleton, and synergistically improve the catalytic activity of ORR.

## 3. Experimental Section

### 3.1. Materials 

Green walnut peel was collected in Linfen, Shanxi Province, China. Melamine can be used directly, which is an analytical grade provided by Sinopharm Chemical (Shanghai, China) Co., Ltd.

### 3.2. Materials Synthesis

Firstly, the green walnut peel is cut into 1 × 1 cm squares, rinsed with deionized water repeatedly, and dried in a constant temperature drying oven at 70 °C for 48 h to obtain dehydrated green walnut peels. It is then crushed with a crusher at 32,000 r/min uniform in 10 min to obtain less than 100 mesh green walnut peel powder, and named GWS precursor (GWS stands for Green walnut skin, the same below). Taken 1 g of GWS precursor and 5 g of melamine (mass ratio 1:5), mixed them evenly, transferred them to high-pressure stainless steel (100 mL) lined with tetrafluoroethylene, and kept them in an oven at 180 °C for 12 h, and the obtained material was named as GWS180M. Then the samples are transferred to a constant temperature oven at 70 °C for drying, and then the GWS180MX was placed in a tube furnace under N_2_ atmosphere. Carbonization is carried out at four given temperatures (700, 800, 900, and 1000 °C) (heating rate is 5 ℃/min, holding time for 4 h). The N-doped carbon material of GWS180M-X (M = Melamine, X represents the carbonization temperature 700, 800, 900, and 1000 °C) was collected at room temperature. All the obtained GWS catalysts were fully ground in a mortar, and soluble impurities were removed by repeated washing with deionized water and absolute ethanol. The cleaned catalysts were dried in an oven at 60 °C.

### 3.3. Structural Characterization

The surface morphology and structure of all samples are measured by a 10 KV field emission scanning electron microscope (SEM, JEOL JSM-7500F, Tokyo, Japan) and a 200 KV transmission electron microscope (TEM, JEOL JEM-2100, Tokyo, Japan). X-ray powder diffraction (XRD) is used to characterize the crystal structure of the catalyst with Cu-Kα radiation in the range of 2θ (10°–80°) with a scan rate of 5°/min. The AMICUS electron spectrometer on SHIMADZU recorded X-ray photoelectron spectroscopy (XPS) using 300 W Al Kα radiation. The electrochemical performance of all catalysts were tested by the electrochemical workstation (Autolab).

## 4. Conclusions

In short, a new type of porous N-doped carbon material can be easily obtained from waste walnut hulls through simple activation and carbonization processes. In this process, using waste walnut hulls as free raw materials has economic and environmental benefits. The resulting material exhibits high electrochemical performance for ORR due to the large surface area and pore structure, as well as isomer doping and abundant defects. The half-wave potential of GWS180M800 after activation and doping with organic pores was greatly increased from 0.70 to 0.82 V, and it showed better stability and methanol resistance than 20 wt% Pt/C. This work provides a new strategy and method for exploring the use of low-cost local biomass materials to produce high value-added free-metal biomass carbon ORR catalysts. 

## Figures and Tables

**Table 1:** Comparison of some advanced metal-free ORR catalysts in 0.1 M KOH electrolyte.

Catalysts	$E_{oneset}\left(V\;vs.\;the\;RHE\right)$	$E_{1/2}\left(V\;vs.\;the\;RHE\right)$	References
PAC-800	0.99 V	0.82 V	4
TARC-N	0.98 V	0.86 V	34
G800-ZC-2.0	0.98 V	0.81 V	33
HC-900	0.95 V	0.80 V	/
N-CSs	0.85 V	0.81 V	/
GWS180M800	1.01 V	0.82 V	This work

## Data Availability

Not applicable.

## Supplementary Materials

The following are available online, Table S1: The content of C, N, and O elements and N configuration calculated of GWS800, GWS180M700, GWS180M800, GWS180M900, and GWS800M1000 from elemental analysis and XPS.

## Sample Availability

Samples of the compounds are available from the authors.
