# Supplementary material for: Nanosheets with High-Performance Electrochemical Oxygen Reduction Reaction Revived from Green Walnut Peel
Source: Molecules. 2022 Jan 5;27(1):328. doi: 10.3390/molecules27010328 (PMC8746947; doi:10.3390/molecules27010328)
Supplement: Supplementary file 1 [file molecules-27-00328-s001.zip › molecules-1525536-supplementary.pdf]

Ultra-thin nanosheets with high-performance electrochemical oxygen reduction reaction derived from green walnut peel

Yifei Zhou[1], Lei Yan[1] and Junhua Hou [1,2,*]

1 School of Physics and Information Engineering, Shanxi Normal University, No.339 Taiyu Road, Xiaodian District, Taiyuan 030031, China; 219110014@sxnu.edu.cn(Y.F.Z); 220110010@sxnu.edu.cn(L.Y.)

2 Extreme Optical Collaborative Innovation Center, Shanxi University, No. 92, Wucheng Road, Xiaodian District, Taiyuan 030006, China

* Correspondence: jhhou@sxnu.edu.cn; Tel.: +86-13203574868

1. Experimental Section

1.1 Synthesis of biomass nanoparticles

The green walnut shells （GWS） peel is first to cut into 1×1 cm squares, rinsed with deionized water repeatedly, and dried in a constant temperature drying oven at 70℃ for 48 hours to obtain dehydrated green walnut peels, and then crushed with a crusher at 32000r/min A uniform 100 mesh green walnut skin powder was obtained in 10 minutes, named GWS precursor (GWS stands for Green walnut skin, the same below), then take a proper amount of GWS precursor and a proper amount of melamine (1:5) and mix it evenly, and transfer to the high-pressure stainless steel lined with tetrafluoroethylene In the reactor (100 ml), use an oven to heat at 180°C for 12h, named GWS180M, then transfer the sample to a constant temperature oven, dry at 70℃, and then take GWS180MX and place it in a tube furnace under N2 atmosphere. Carbonization is carried out at four given temperatures (700, 800, 900, and 1000℃). The heating rate is 5℃/min, and the heating is 4 hours. The N-doped carbon material of GWS180M-X (M=Melamine, X represents the carbonization temperature 700, 800, 900, and 1000℃) was collected at room temperature. Finally, a series of GWS materials were fully ground in an agate mortar, and soluble impurities were repeatedly washed with deionized water and absolute ethanol and dried at 60℃.

The GWS-X samples were characterized by the Raman, SEM and TEM measurements. Some of the material characterization and equipment

are the same as the previous reports.

1.2 Evaluation of the electrocatalytic activity toward ORR

To prepare the electrode for ORR measurements, some GWS-X nanoparticles were dispersed in the solution of $50\mu L$ Nafion solution, $250\mu L$ isopropanol, and 700 μLdeionized water were mixed in a $1.5mL$ centrifuge tube and sonicated for 1 hour using a sonicator to form a catalyst suspension. Finally, take 10 μLdroplets of the catalyst suspension on the surface of the electrode and wait for it to dry naturally (the average catalyst loading is $0.25mg.cm^{-2}$).

The electrocatalytic activity was measured in a 0.1 M oxygen saturated KOH solution using an electrochemical workstation (CHI 760E, CH Instruments Inc., Shanghai, China) in a standard three electrode system. The three-electrode system was a catalyst-modified glass rotating disc electrode (GC-RDE) as the working electrode, with platinum wire and a 3M/L KCl solution The Ag/AgCl electrodes are the counter electrode and the reference electrode. All measured potentials are referred to as reversible hydrogen electrodes (RHE) in 0.1 M KOH by RHE calibration, as shown in the following equation:

$$E_{RHE} = E_{Ag/AgCl} + 0.9762V \quad (1)$$

Linear sweep voltammetry (LSV) was carried out at a scan rate of 1.0 mV s^{-1} for the whole polarization curves.

Table S1. The content of C, N, and O elements and N configuration calculated of GWS800, GWS180M700, GWS180M800, GWS180M900, and GWS800M1000 from elemental analysis and XPS.

Samples	Elemental content (at. %)			N configuration (%)			
	C 1s	N 1s	O 1s	pyridinic-N	pyrrolic-N	graphitic-N	oxidized-N
GWS800	81	0.87	14.23	24.1	4.1	20.5	51.2
GWS180M700	73.91	2.11	9.59	37.9	6.6	44.0	9.3
GWS180M800	70.98	10.46	16.73	48.5	30.6	19.0	1.9
GWS180M900	76.46	5	14.4	35.9	44.2	15.4	4.4
GWS180M1000	79.18	1.28	13.55	11.7	83.3	1.7	3.3
